# Correlation between Common Lower Genital Tract Microbes and High-Risk Human Papillomavirus Infection

**DOI:** 10.1155/2019/9678104

**Published:** 2019-11-22

**Authors:** Panpan Lv, Fang Zhao, Xiaoqin Xu, Jun Xu, Qiang Wang, Zhen Zhao

**Affiliations:** ^1^Department of Clinical Laboratory, Minhang Hospital, Fudan University, No. 170 XinSong Road, Shanghai 201199, China; ^2^Department of Obstetrics and Gynecology, Minhang Hospital, Fudan University, No. 170 XinSong Road, Shanghai 201199, China

## Abstract

**Background:**

High-risk human papillomavirus (hr-HPV) infection is a necessary cause of cervical cancer. However, other common lower genital tract microbes may increase hr-HPV infection and their related cervical cytopathy.

**Methods:**

To confirm this hypothesis, cervical brush and vaginal swab specimens were collected from 826 adult patients who were divided into the hr-HPV-positive group (254) and the negative group (572) by real-time PCR assay. Cervical specimens were tested for *Ureaplasma parvum* (UP), *Ureaplasma urealyticum* (UU), and *Chlamydia trachomatis* (CT) using PCR analysis. Vaginal secretion was detected for *Trichomonas vaginalis* (TV), *Candida* spp., and bacterial vaginosis (BV) with conventional assay.

**Results:**

Among hr-HPV-positive women, UP was found in 51.6%, UU in 15.4%, CT in 15.7%, *Candida* spp. in 11.0%, TV in 3.1%, and BV in 20.5%. In the hr-HPV-negative group, UP was positive in 36.2%, UU in 8.6%, CT in 4.0%, *Candida* spp. in 12.4%, TV in 0.2%, and BV in 7.0%. Multivariate logistic regression analysis with age-adjusted showed that UU (OR, 1.757), UP (OR, 1.804), CT (OR, 3.538), BV (OR, 3.020), and TV (OR, 14.109) were risk factors on hr-HPV infection (*P* < 0.05).

**Conclusion:**

These microbes might induce cervical chronic inflammation that would damage the mucosal barrier and immune protection to promote the infection of hr-HPV.

## 1. Introduction

Genital hr-HPV infection is considered as a necessary cause of cervical cancer [1], which is the fourth most common neoplasm among women worldwide, affecting about 530 thousand women every year [2]. Hr-HPV specific genotypes, viral load, and coinfection of HPV multiple genotypes are important factors for the development of cervical cytopathology [3]. And many other cofactors are involved in this cytopathological process, such as long-term use of hormonal contraceptives, high parity, smoking, immunosuppression, and certain dietary deficiencies [3]. In addition, the sexually transmitted pathogens were reported to interact with HPV as cofactors in the progression of cervical neoplasias. For instance, C*hlamydia trachomatis* (CT) and *Trichomonas vaginalis* (TV) were proved to be positively correlated with hr-HPV persistent infection [4, 5]. Vielot et al. [6] also reported that CT was a significant risk factor for hr-HPV long-term infection. A meta-analysis from 22 studies with 16,181 women showed that *Ureaplasma* spp. was associated with hr-HPV infection and the abnormal cervical cytopathology [7]. However, the above results remain inconclusive. The aim of this research was to reveal the correlation between other common lower genital tract microbes and hr-HPV infection, including *Chlamydia trachomatis*, *U. urealyticum* (UU), *Ureaplasma parvum* (UP), *Trichomonas vaginalis*, and *Candida* spp. Moreover, bacterial vaginosis (BV) was also investigated.

## 2. Study Design

### 2.1. Selection of Subjects

A total of 826 women patients who attended the outpatient clinic of Minhang Hospital, Fudan University (Shanghai, China), between January and May 2019 were enrolled in this study. The selection of participants was conducted by consecutive sampling. The inclusion criteria were as follows: the women had sexual experience; aged between 20 and 70. Exclusion criteria were pregnancy, lower genital tract dysplasia or malignancy, history of cervical cancer, had been treated with vaginal medication in the previous 3 days, and other acute or chronic nongynecological diseases [8, 9]. This study was approved by the Ethics Committee of Minhang Hospital, Fudan University. A written informed consent was obtained from all participants.

### 2.2. HPV-DNA Test

Cervical exfoliated cells were collected using the cytobrush and placed in 2 ml liquid-based cytology medium (Jianyou Medical Tech Co. Ltd, Jiangsu, China). The uterine orifice would be cleaned with a sterile cotton swab when there was excessive mucus and exudates. The specimens were extracted and detected for hr-HPV DNA using the cobas® 4800 System (Roche Molecular Systems, Inc., Branchburg, USA) that fully automated sample preparation and real-time polymerase chain reaction (PCR) amplification of 14 hr-HPV L1 genes (HPV-16, -18, -31, -33, -35, -39, -45, -51, -52, -56, -58, -59, -66, and -68). TaqMan probes were labeled with 4 different fluorescent dyes for HPV-16, HPV-18, the other 12 hr-HPV, and the human *β*-globin gene, respectively. The human *β*-globin gene was used to monitor the whole detection process as a quality control [10].

### 2.3. Detection of BV, TV, and *Candida* spp

The vaginal swabs samples were collected from the posterior vaginal fornix and placed in 0.5 ml of sterile physiologic saline. TV and *Candida* spp. were detected using microscopic examination (100× and 400×) within 15 min. [11] *Candida* spp. budding cells and hyphae were detected by Gram staining. BV was diagnosed based on Nugent's criteria using the results from a Gram-stained slide [12]. A Nugent score ≥7 was defined as BV. All samples were evaluated independently by two experienced cytotechnologists, and the final results were established based on consensus of both.

### 2.4. Detection of UU, UP, and CT DNA

Cervical specimens were collected for detection of UU, UP, and CT genes. CT DNA was extracted by genome extraction kits and amplified by real-time PCR kits according to the manufacturer's instructions (Fosun Pharma Co. Ltd, Shanghai, China). UU and UP detection was performed using a previously published real-time PCR assay described by Liu et al. [13]. To prevent contamination of the amplified products, dUTP and UNG (uracil-N-glycosylase) enzymes were used. In addition, an exogenous internal process control (glyceraldehyde-3-phosphate dehydrogenase gene) was performed to monitor and avoid the “false negative” results caused by PCR inhibitors in the sample.

### 2.5. Statistical Analysis

All statistical analyses were performed using R software version 3.5.1. Patient ages were expressed as mean ± standard deviation (SD), with differences tested by *t*-test. Hr-HPV prevalence among different age groups was analyzed with chi-square (*χ*^2^) test. Multivariable logistic regression was used to determine the correlation of hr-HPV infection and other microorganisms (UU, UP, CT, *Candida* spp., TV, and BV). The crude relative risk ratio (OR) with the corresponding 95% confidence intervals (CI) and the age-adjusted relative risk ratio were used to calculate the risk. A *P* value <0.05 was considered statistically significant.

## 3. Results

### 3.1. HPV Infection Status

Of 826 enrolled women, hr-HPV-positive patients were 254 (30.8%) with an average age of 37.7 ± 12.3 and HPV-negative women were 572 with an average age of 39.3 ± 10.7. There was no statistically significant difference in the composition of age between two groups (*p*=0.0681). However, hr-HPV prevalence had significant differences among different age groups (*χ*^2^ = 29.848, *p*=0.001). A higher prevalence of hr-HPV was showed in the group <30 years old (45.2%) and the group ≥60 years old (41.1%). The hr-HPV-positive women herein showed a comparatively younger and higher age, of which proportionately more of them were sexually active and postmenopausal, Table 1.

### 3.2. Prevalence of Hr-HPV and Other Lower Genital Tract Microbes

Of 826 women, hr-HPV was detected in 254. Genotypes were showed as follows: HPV-16 was positive in 97 (11.7%), HPV-18 in 22 (2.7%), and the other 12 hr-HPV genotypes in 135 (16.3%). UP, UU, CT, *Candida* spp., TV, and BV were positive in 338 samples (40.9%), 88 samples (10.7%), 63 samples (7.6%), 99 samples (12.0%), 9 samples (1.1%), and 92 samples (11.1%), respectively. Among the above lower genital tract microbes, UP was the most common one (*P* < 0.05).

### 3.3. Coinfection of Hr-HPV and Other Lower Genital Tract Microbes

In the hr-HPV-positive group, UP was found in 51.6%, UU in 15.4%, CT in 15.7%, *Candida* spp. in 11.0%, TV in 3.1 %, and BV in 20.5%. Among the hr-HPV-negative women, UP was present in 36.2%, UU in 8.6%, CT in 4.0%, *Candida* spp. in 12.4%, TV in 0.2%, and BV in 7.0% (Table 2). The prevalence of UP, UU, CT, TV, and BV in hr-HPV-positive group was all significantly higher than that in the HPV-negative group (*P* < 0.05). But the prevalence of *Candida* spp. was not significantly different between the two groups (*P*=0.505). The data were evaluated using multivariate logistic regression analysis that was adjusted for age alone. And it showed that UU (OR, 1.757), UP (OR, 1.804), CT (OR, 3.538), BV (OR, 3.020), and TV (OR, 14.109) were risk factors of hr-HPV infection (*P* < 0.05).

## 4. Discussion

So far, more than 200 HPV genotypes have been characterized. Some of them infect the genital tract and have higher oncogenic potential known as hr-HPVs. Considering the correlation between hr-HPV and cervical cancer, it will be useful to identify those patients with hr-HPV infection and may therefore provide valuable information for their prevention and treatment. In this study, 14 hr-HPV genotypes were tested in specimens from 826 patients, of which 30.8% showed positive results. It was higher than that in the health checkups (10.0%–14.9%) and similar with gynecological outpatients (20.6%–31.5%), but lower than that in the patients with cervical cytopathy (59.6%–84.8%) reported from Li et al. [14]. This suggested that there might be a correlation between hr-HPV infection and cervical cytopathy. As shown in our previous study on screening performance of 14 hr-HPV types testing in cervical lesions, the positive rate of hr-HPV increased with the severity of cervical lesion (*P*=0.001). A study conducted by Kim et al. [15] showed the same conclusion. In addition to the significant correlations between hr-HPV and abnormal cervical cytology, the total sexually transmitted infections (*Chlamydia trachomatis, Mycoplasma genitalium, Neisseria gonorrhoeae, Mycoplasma hominis,* TV, UU, and UP) positive rate was significantly higher when a cytological diagnosis was a grade equal to or higher than atypical squamous cells of undetermined significance (ASC-US) among the hr-HPV-negative group.

The age-specific prevalence curve showed that hr-HPV infections peaked firstly in women younger than 30 years, and then it declined with advancing age, but the curve rose again at age 50 years and peaked at age 60 years or older. It was similar with the reports from Herrero et al. [16]. The first peak in the youngest women may be correlated with the high sexual activity or multiple sex partners. Many researchers found that most HPV infections became undetectable within 1-2 years. Presumably, the virus was cleared by the host's cellular immunity or was suppressed into long-term latency [16, 17]. The second prevalent peak was in postmenopausal women, which had not been noted by other researchers. It may be because their data did not include enough older women. The possible explanation for the second peak would be that older women had been exposed more intensely to HPV, decreased immune clearance, and reactivation of a latent HPV infection [16]. The future studies should focus on markers of immune suppression, tests for activation of latent HPV, and the sexual practice of older women and their male partners.

To explore the correlation between common lower genital tract microbes and HPV infection, UU, UP, CT, TV, *Candida* spp., and BV were detected in 826 cervical and vaginal specimens. Except *Candida* spp., these microbes all showed higher prevalence in HPV-positive women than HPV-negative women. Multiple regression analysis indicated that UU, UP, CT, TV, and BV were all risk factors for hr-HPV infection.

Previous study had demonstrated that vaginal colonization with *Ureaplasma* spp. among women was 40–80% [18]. And a high density of *Ureaplasma* spp. seemed to be correlative with the development of HPV infection and cervical lesions [8, 19]. In our study, *Ureaplasma* spp. was differentiated into UU and UP, both of which showed a higher prevalence in hr-HPV infected women and were related to hr-HPV status. A similar result was found in healthy Italian women by Camporiondo et al. [20]. In their study, a significant association was showed between HPV infection and presence of UU or UP (*P* < 0.05). However, another study from Zhang et al. [9] did not support a positive association between *Ureaplasma* spp. infection and hr-HPV status. But they also speculated that *Ureaplasma* spp. might play a role in HPV persistence and initiating cellular anomalies. These differences might come from sampling differences in research population and needed to be confirmed through a multicenter randomized controlled study in the future. UU has been reported to be associated with genital diseases and aroused a widespread concern. Instead, UP was known as a normal flora of the women lower genital tract, and its harmfulness was often overlooked. However, it was indicated that *Ureaplasma* spp. infections, especially their chronic inflammation development, might favor the entry of other microorganisms, act as cofactor in the pathogenesis of cervical disease, and induce chromosomal alterations that might lead to carcinogenesis of epithelial cells [7, 20–23].

The correlation between CT and HPV infection had been established by others. Our data also revealed about 4-fold higher risk of hr-HPV infection in CT-positive women compared with CT-negative group, indicating the necessity of screening and treatment for CT in hr-HPV-positive women. It was suggested that CT infection could increase cervical susceptibility to HPV by inflammation and epithelial cytopathy thus facilitating the entry of virions; considering the immune response was crucial for the clearance of HPV infection. The chronic cervical inflammation could reduce host cell-mediated immunity by raising production of free radicals, which seemed to be favourable to the persistence of HPV [8]. CT infection had been demonstrated as a public health problem, which may damage the mucosal barrier, allow HPV to penetrate into the basal epithelium layer, interfere immune response, and increase the infection of HPV and the risk of carcinogenesis [24]. Some previous studies had verified an increased CT infection and coinfection of CT with HPV in sexually active women. Additionally, women with CT infection had a nearly two-fold duration of hr-HPV infection, providing extra support to the correlation between CT and HPV persistence [6, 8, 24].

It was reported that TV could induce inflammation in the cervicovaginal epithelium, disturb the epithelium integrity, facilitate virus access to underlying layers, and result in HPV persistent infection [25]. Ghosh et al. [26] found that women coinfected with TV and hr-HPV had higher risk of invasive cervical cancer. As well, a significant association between HPV-16 and TV was revealed by a research on 324 patients from rural Tanzania. Women infected with TV were 6.5 times more likely to get HPV-16 compared with women without TV (50% vs 13.3%) [27]. Our data also found a 14.109-fold increased risk of hr-HPV infection in TV-positive women than TV-negative ones, showing a strong correlation between TV and 14 hr-HPV genotypes infection.

In this study, BV was also found to be associated with hr-HPV infection. It was consistent with the reports from Zhang et al. [9] BV is characterized as alterations in the vaginal flora: a decrease in *Lactobacillus* spp. and a concomitant increase of facultative anaerobes or anaerobic bacteria, including *Gardnerella vaginalis*, *Prevotella* species, and *Porphyromonas* species. BV could affect cytokines and chemokines, antimicrobial proteins, and immune cell populations in the vagina, resulting in tissue damage and enhancing the oncogenic potential of HPV [28]. And according to King et al., [29] the clearance of HPV was delayed among women who had BV, leading to the increasing HPV events. Subsequently, cervical dysplasia and neoplastic lesions were eventually developed. Also they demonstrated that BV, whatever in previous or current infection, was associated with incident HPV infections. Since BV could bring about major changes in the vaginal environment and cause innate defenses degradation, it was plausible to pay attention to the disturbed bacterial ecologic system to control the incidence of HPV and cervical carcinogenesis.

Our study has some limitations. First of all, the study population only includes gynecological clinic patients, but not women who had physical examinations. And there are many other microbes (*Neisseria gonorrhoeae, Mycoplasma hominis, Mycoplasma genitalium*, herpes virus, etc.) in the lower genital tract that were not included in this study. To overcome the shortcoming, a multicenter, large-sample, case-control study will be needed in the future. Secondly, some of the testing methods are not novel enough in this research, such as microscopic examination was used to diagnose *Candida* spp. and TV. To make up for these deficiencies and to ensure the accuracy of test results, we would replace microscopic examination with PCR or other confirmatory gold standard technique in our future study. Finally, only partial participants filled out the questionnaire about menopause status, smoking, contraception, and oral contraceptives. They all had no statistically significant correlation with hr-HPV infections, which was in consistent with other studies [27, 30]. Sexual behavior is an important factor that can increase the risk of HPV as well as other microbes, which we had not included in this research. The above information, as well as socioeconomic and lifestyle factors, infections persistence, medical treatment, and disease progression should be included in the future study, so as to comprehensively and systematically evaluate the risk factors for HPV infection and cervical cytopathy.

In conclusion, the high prevalence of UU, UP, CT, TV, and BV were found in hr-HPV-positive women, which were showed as risk factors of hr-HPV infections. These lower genital tract microbes could induce cervical inflammation, increase free radical generation, damage cervical epithelial barrier, and reduce immune clearance, so as to promote the infection of hr-HPV. It may be useful to screen these microbes in adult women.

## Figures and Tables

**Table 1:** Hr-HPV infection status among patients within different age groups.

	HPV+	HPV−	HPV (+)%	*P*
Age (continuous)				
Mean ± SD	37.7 ± 12.3	39.3 ± 10.7		0.0681
Age group (years)				0.001^*∗*^
<30	84	102	45.2	
30–39	77	222	25.8	
40–49	46	149	23.6	
50–59	24	66	26.7	
≥60	23	33	41.1	

HPV: high-risk human papillomavirus; ^*∗*^significant difference as *P* < 0.05.

**Table 2:** Multivariate logistic regression analysis of the association between hr-HPV status and other microorganisms.

Variables	*n*	hr-HPV status		Crude relative risk ratio	Age-adjusted relative risk ratio	*P* value
		Negative	Positive	OR (95% CI)	OR (95% CI)	
*Ureaplasma urealyticum*						0.020^*∗*^
Negative	738	523	215	Reference	Reference	
Positive	88	49	39	1.766 (1.082–2.859)	1.757 (1.076–2.848)	

*Ureaplasma parvum*						<0.001^*∗*^
Negative	488	365	123	Reference	Reference	
Positive	338	207	131	1.834 (1.334–2.524)	1.804 (1.309–2.489)	

*Chlamydia trachomatis*						<0.001^*∗*^
Negative	763	549	214	Reference	Reference	
Positive	63	23	40	3.600 (2.073–6.363)	3.538 (2.033–6.268)	

*Candida* spp.						0.505
Negative	727	501	226	Reference	Reference	
Positive	99	71	28	0.799 (0.481–1.295)	0.788 (0.474–1.279)	

*Trichomonas vaginalis*						0.012^*∗*^
Negative	817	571	246	Reference	Reference	
Positive	9	1	8	14.609 (2.546–275.946)	14.109 (2.463–266.483)	

*Bacterial vaginosis*						<0.001^*∗*^
Negative	734	532	202	Reference	Reference	
Positive	92	40	52	3.024 (1.901–4.831)	3.020 (1.899–4.825)	

^*∗*^Significant difference as *P* < 0.05; OR, odds ratio.

## Data Availability

The data used to support the findings of this study are available from the corresponding author upon request.

## References

[1] Zur Hausen H. (2002). Papillomaviruses and cancer: from basic studies to clinical application. *Nature Reviews Cancer*.

[2] Taylor S., Bunge E., Bakker M. (2016). The incidence, clearance and persistence of non-cervical human papillomavirus infections: a systematic review of the literature. *BMC Infectious Diseases*.

[3] Muñoz N., Castellsagué X., de González A. B., Gissmann L. (2006). Chapter 1: HPV in the etiology of human cancer. *Vaccine*.

[4] Samoff E., Koumans E. H., Markowitz L. E. (2005). Association of *Chlamydia trachomatis* with persistence of high-risk types of human papillomavirus in a cohort of female adolescents. *American Journal of Epidemiology*.

[5] Shew M. L., Fortenberry J. D., Tu W. (2006). Association of condom use, sexual behaviors, and sexually transmitted infections with the duration of genital human papillomavirus infection among adolescent women. *Archives of Pediatrics & Adolescent Medicine*.

[6] Vielot N., Hudgens M. G., Mugo N., Chitwa M., Kimani J., Smith J. (2015). The role of *Chlamydia trachomatis* in high-risk human papillomavirus persistence among female sex workers in Nairobi, Kenya. *Sexually Transmitted Diseases*.

[7] Ye H., Song T., Zeng X., Li L., Hou M., Xi M. (2018). Association between genital mycoplasmas infection and human papillomavirus infection, abnormal cervical cytopathology, and cervical cancer: a systematic review and meta-analysis. *Archives of Gynecology and Obstetrics*.

[8] Verteramo R., Pierangeli A., Mancini E. (2009). Human Papilloma viruses and genital co-infections in gynaecological outpatients. *BMC Infectious Diseases*.

[9] Zhang D., Li T., Chen L., Zhang X., Zhao G., Liu Z. (2017). Epidemiological investigation of the relationship between common lower genital tract infections and high-risk human papillomavirus infections among women in Beijing, China. *PLoS One*.

[10] Rao A., Young S., Erlich H. (2013). Development and characterization of the cobas human papillomavirus test. *Journal of Clinical Microbiology*.

[11] Liu J., Liu W., Liu Y., Zhou X., Zhang Z., Sun Z. (2016). Prevalence of microorganisms co-infections in human papillomaviruses infected women in Northern China. *Archives of Gynecology and Obstetrics*.

[12] Nugent R. P., Krohn M. A., Hillier S. L. (1991). Reliability of diagnosing bacterial vaginosis is improved by a standardized method of gram stain interpretation. *Journal of Clinical Microbiology*.

[13] Liu L., Ji Y. H., Zhao Z. (2011). Clinical application of genotyping detection of human Mycoplasma urealyticum based on parC gene sequences. *Chinese Journal of Microbiology and Immunology*.

[14] Li K., Li Q., Song L., Wang D., Yin R. (2019). The distribution and prevalence of human papillomavirus in women in mainland China. *Cancer*.

[15] Kim H.-S., Kim T. J., Lee I.-H., Hong S. R. (2016). Associations between sexually transmitted infections, high-risk human papillomavirus infection, and abnormal cervical Pap smear results in OB/GYN outpatients. *Journal of Gynecologic Oncology*.

[16] Herrero R., Hildesheim A., Bratti C. (2000). Population-based study of human papillomavirus infection and cervical neoplasia in rural Costa Rica. *JNCI: Journal of the National Cancer Institute*.

[17] Schiffman M., Kjaer S. K. (2003). Chapter 2: natural history of anogenital human papillomavirus infection and neoplasia. *JNCI Monographs*.

[18] Waites K. B., Katz B., Schelonka R. L. (2005). Mycoplasmas and ureaplasmas as neonatal pathogens. *Clinical Microbiology Reviews*.

[19] Lukic A., Canzio C., Patella A. (2006). Determination of cervicovaginal microorganisms in women with abnormal cervical cytology: the role of *Ureaplasma urealyticum*. *Anticancer Research*.

[20] Camporiondo M. P., Farchi F., Ciccozzi M. (2016). Detection of HPV and co-infecting pathogens in healthy Italian women by multiplex real-time PCR. *Le Infezioni in Medicina*.

[21] Choi Y., Roh J. (2014). Cervical cytopathological findings in Korean women with *Chlamydia trachomatis*, Mycoplasma hominis, and *Ureaplasma urealyticum* infections. *Scientific World Journal*.

[22] Zhang S., Wear D. J., Lo S. (2000). Mycoplasmal infections alter gene expression in cultured human prostatic and cervical epithelial cells. *FEMS Immunology and Medical Microbiology*.

[23] Zhang S., Tsai S., Lo S. C. (2006). Alteration of gene expression profiles during mycoplasma-induced malignant cell transformation. *BMC Cancer*.

[24] Silva J., Cerqueira F., Medeiros R. (2014). *Chlamydia trachomatis* infection: implications for HPV status and cervical cancer. *Archives of Gynecology and Obstetrics*.

[25] Costa R. F. M. d., Souza W. d., Benchimol M., Alderete J. F., Morgado-díaz J. A. (2005). Trichomonas vaginalis perturbs the junctional complex in epithelial cells. *Cell Research*.

[26] Ghosh I., Muwonge R., Mittal S. (2017). Association between high risk human papillomavirus infection and co-infection with *Candida* spp. and *Trichomonas vaginalis* in women with cervical premalignant and malignant lesions. *Journal of Clinical Virology*.

[27] Lazenby G. B., Taylor P. T., Badman B. S. (2014). An association between *Trichomonas vaginalis* and high-risk human papillomavirus in rural Tanzanian women undergoing cervical cancer screening. *Clinical Therapeutics*.

[28] Onderdonk A. B., Delaney M. L., Fichorova R. N. (2016). The human microbiome during bacterial vaginosis. *Clinical Microbiology Reviews*.

[29] King C. C., Jamieson D. J., Wiener J. (2011). Bacterial vaginosis and the natural history of human papillomavirus. *Infectious Diseases in Obstetrics and Gynecology*.

[30] Mongelos P., Mendoza L. P., Rodriguez-Riveros I. (2015). Distribution of human papillomavirus (HPV) genotypes and bacterial vaginosis presence in cervical samples from Paraguayan indigenous. *International Journal of Infectious Diseases*.

